# Deep expression analysis reveals distinct cold-response strategies in rubber tree (*Hevea brasiliensis*)

**DOI:** 10.1186/s12864-019-5852-5

**Published:** 2019-06-04

**Authors:** Camila Campos Mantello, Lucas Boatwright, Carla Cristina da Silva, Erivaldo Jose Scaloppi, Paulo de Souza Goncalves, W. Brad Barbazuk, Anete Pereira de Souza

**Affiliations:** 1 Molecular Biology and Genetic Engineering Center (CBMEG), University of Campinas (UNICAMP), Campinas, SP Brazil; 2 Department of Biology, University of Florida, Gainesville, FL USA; 3 The John Bingham Laboratory, National Institute of Agricultural Botany, Cambridge, UK; 4 Rubber Research Advanced Center (CAPSA), Agronomical Institute (IAC), Votuporanga, SP Brazil; 5 Genetics Institute, University of Florida, Gainesville, FL USA; 6 Department of Plant Biology, Biology Institute, University of Campinas (UNICAMP), Campinas, SP Brazil

**Keywords:** *Hevea brasiliensis*, RNA-seq, Transcriptome, Gene expression, Alternative splicing, Cold stress, Molecular marker, Microsatellite, Single nucleotide polymorphism

## Abstract

**Background:**

Natural rubber, an indispensable commodity used in approximately 40,000 products, is fundamental to the tire industry. The rubber tree species *Hevea brasiliensis* (Willd. ex Adr. de Juss.) Muell-Arg., which is native the Amazon rainforest, is the major producer of latex worldwide. Rubber tree breeding is time consuming, expensive and requires large field areas. Thus, genetic studies could optimize field evaluations, thereby reducing the time and area required for these experiments. In this work, transcriptome sequencing was used to identify a full set of transcripts and to evaluate the gene expression involved in the different cold-response strategies of the RRIM600 (cold-resistant) and GT1 (cold-tolerant) genotypes.

**Results:**

We built a comprehensive transcriptome using multiple database sources, which resulted in 104,738 transcripts clustered in 49,304 genes. The RNA-seq data from the leaf tissues sampled at four different times for each genotype were used to perform a gene-level expression analysis. Differentially expressed genes (DEGs) were identified through pairwise comparisons between the two genotypes for each time series of cold treatments.

DEG annotation revealed that RRIM600 and GT1 exhibit different chilling tolerance strategies. To cope with cold stress, the RRIM600 clone upregulates genes promoting stomata closure, photosynthesis inhibition and a more efficient reactive oxygen species (ROS) scavenging system. The transcriptome was also searched for putative molecular markers (single nucleotide polymorphisms (SNPs) and microsatellites) in each genotype. and a total of 27,111 microsatellites and 202,949 (GT1) and 156,395 (RRIM600) SNPs were identified in GT1 and RRIM600. Furthermore, a search for alternative splicing (AS) events identified a total of 20,279 events.

**Conclusions:**

The elucidation of genes involved in different chilling tolerance strategies associated with molecular markers and information regarding AS events provides a powerful tool for further genetic and genomic analyses of rubber tree breeding.

**Electronic supplementary material:**

The online version of this article (10.1186/s12864-019-5852-5) contains supplementary material, which is available to authorized users.

## Background

Cold stress, which can be classified as chilling (0 to 15 °C) and/or freezing (< 0 °C) temperatures, affects plant growth and development, limiting spatial distribution and yields [1, 2]. Furthermore, cold stress prevents plants from achieving their full genetic potential, inhibiting metabolic reactions, reducing the photosynthetic capacity and altering membrane permeability [1, 3].

Temperate plants can generally achieve cold acclimation and acquire tolerance to extracellular ice formation in their vegetative tissues. However, tropical crops such as maize and rice lack the cold acclimation ability and are sensitive to chilling [1]. Furthermore, varieties from the same species can exhibit different levels of cold tolerance [4, 5]. Hence, determining gene expression profiles under cold stress could help to elucidate the mechanisms of cold acclimation in plants and can be an effective method for selecting candidate genes. Moreover, candidate genes can be targeted to identify genetic variation and develop molecular markers.

*Hevea brasiliensis* [(Willd. ex Adr. de Juss.) Muell-Arg], commonly known as the rubber tree, is a perennial tree crop native to the Amazon rainforest. This species, which belongs to the Euphorbiaceae family, is monoecious, undergoes cross-pollination and has a chromosome number of 2*n* = 2x = 36. Among the 2500 species that produce natural rubber (cis-1,4-polyisoprene), *H. brasiliensis* is the only species that produces high-quality rubber in commercially viable quantities, accounting for more than 98% of total worldwide rubber production [6].

Natural rubber is one of the most important raw materials for many industries and cannot be replaced by synthetic alternatives due to its unique properties, such as flexibility, elasticity and abrasion resistance [7]. Natural rubber is an essential commodity for the tire industry and for the manufacture of more than 40,000 different products.

Although the Amazon basin offers a suitable climate for this crop, Southeast Asia is the major producer of rubber responsible for 92% of the worldwide rubber production. South America is responsible for only 2% of worldwide rubber production due to the occurrence of the fungus *Microcyclus ulei* (P. Henn) v. Arx, which causes South American leaf blight (SALB). SALB devastated plantations in northern Brazil in the 1930s and remains a permanent threat to the rubber industry [8]. To date, rubber tree plantations in Southeast Asia have not been affected by SALB, but other native pathogenic fungi are threats to rubber production. The two major fungal pathogens in Southeast Asia (*Phytophthora* and *Corynespora*) cause leaf fall and, consequently, significant losses of natural rubber yields [6]. Due to the occurrence of diseases, plantations have been expanded to the suboptimal areas of some countries, such as northeastern India, the highlands and coastal areas of Vietnam, southern China and the southern plateau of Brazil [9]. These areas are characterized by new stress conditions, such as cold and dry periods.

The exposure of rubber trees to low temperatures can cause leaf necrosis, which affects tree development and latex production [9, 10]. For young rubber plants, low temperatures can cause death. In addition, low temperatures are responsible for halting latex production for 1–3 months per year [11, 12]. For this reason, breeding programs are interested in clones that show continuous increment and latex production under suboptimal conditions.

The physiological response to study different strategies to cope with cold stress was previously studied in eight commercial rubber tree clones. Among these clones, only the RRIM600 clone was resistant, showing no leaf damage after cold exposure, while three other clones (GT1 YUNYAN7–4 and IRCA707) were classified as tolerant because they presented few leaf injuries. This study suggests that RRIM600 uses an avoidance strategy with fast stomata closure, the downregulation of photosynthetic activity and strong CO2 assimilation inhibition after chilling stress, while the tolerant genotypes still demonstrate photosynthetic activity and remain active during chilling treatment. Interestingly, the strategy used by RRIM600 restricts permanent damage but impairs growth in chilling conditions. [10].

In recent years, there has been an exponential increase of genomic data for rubber tree, including transcriptome profiles [13–15], linkage maps [16–18] and, more recently, a genome assembly [6, 19]. A recent gene expression study in rubber tree using RNA-seq data evaluated the responses of cold-tolerant and cold-susceptible clones. The authors suggested that the cold-tolerant genotype downregulated auxin and ethylene signaling genes under cold stress, while the heat shock module and reactive oxygen species (ROS) scavengers were upregulated and represented the primary strategy for coping with cold temperatures [20].

To understand the genetic cold tolerance mechanism, we conducted a chilling stress experiment (10 °C) with the clones RRIM600 and GT1, previously described as cold resistant and cold tolerant [10], respectively; these clones exhibit high yields and are recommended for planting in escape areas. RNA sequencing was performed with the aim of constructing a comprehensive transcriptome and investigating the differentially expressed genes (DEGs) involved in different cold acclimation strategies. In addition, the comprehensive transcriptome was searched for putative molecular markers (single nucleotide polymorphisms (SNPs) and microsatellites) and to detect alternative splicing (AS) events.

## Results

### Sequencing and transcriptome assembly

In the present study, we sequenced leaf tissue RNA from the RRIM600 and GT1 genotypes, which resulted in a total of 529,339,330 paired-end (PE) reads for the RRIM600 genotype and 632,887,764 PE reads for the GT1 genotype. After removing low-quality reads, the cDNA libraries from RRIM600 yielded 432,005,062 (81.6%) high-quality (HQ) PE reads, while those from GT1 yielded 501,609,042 (79.2%) HQ PE reads. When we summarized the total HQ PE reads from both genotypes, we obtained 933,614,104 reads, which were employed to construct the reference transcriptome.

To generate a comprehensive transcriptome, we used the Program to Assemble Spliced Alignments (PASA) [21] pipeline to update and maximize the recovery of gene structure and spliced isoforms. A total of 39,351 nonredundant ESTs from NCBI were combined with the 335,212 transcripts obtained in the genome-guided assembly and the 114,718 filtered transcripts obtained from the genome annotation (Additional file 1: Material) and then aligned against the rubber tree genome [19].

A total of 250,458 transcripts, which clustered with 162,278 genes, were obtained. The N50 was 2095 bp, and the GC content was 40.31%. Transcripts were filtered out according to their length (≤ 500 bp), their BLASTX-determined similarity to nonplant sequences and whether the genome annotation was the only evidence of the prediction. After filtering the transcripts using PASA, a total of 104,738 transcripts were clustered with 49,304 genes (Table 1). The total number of predicted genes in this study was similar to the prediction of Tang et al. [19] for the rubber tree genome (43,792 genes). The N50 of the reference transcriptome obtained in this study was 2369 bp with a GC% content of 40.16% (Table 1). Of the total transcripts, 37,302 (35.6%) ranged in size from 1000 bp to 1999 bp, and 36,681 (35%) of the transcripts were longer than 2 kb. The 104,738 transcripts were considered a good reference transcriptome and employed for further analysis.

**Table 1:** Summary statistics for the comprehensive transcriptome

Total number of contigs	104,738
Total number of genes	49,304
Total number of nucleotides	196,309,369
Min contig length (bp)	500
Max contig length (bp)	22,333
Mean contig length (bp)	1874
N50	2369
GC content	40.16%

### Functional annotation

In total, 63,983 (61%) transcripts were annotated against the SwissProt/UniProt database, and among the 94,166 proteins predicted with Transdecoder, 42.262 (44.9%) were annotated.

The Pfam annotation contained protein domains for 67,628 (71.8%) predicted proteins. The top 20 protein domains are presented in Fig. S1. The most abundant type of protein domain was the protein kinase domain, which is a superfamily that is involved in responses to many signals, such as light, hormones and temperature stress [22]. The next three most abundant gene families identified were the protein tyrosine kinase, leucine-rich repeat (LRR) N-terminal domain and NB-ARC domain families (Fig. S1). The protein tyrosine kinase family is responsible for signal transduction in plants in response to stress and developmental processes [21]. Proteins containing the LRR N-terminal domain are involved in numerous functions, such as signal transduction, cell adhesion, DNA repair, disease resistance, apoptosis and the immune response. The NB-ARC domain is a functional ATPase, and its nucleotide-binding state regulates the activity of resistance (R) proteins, which are involved in pathogen recognition and activate the immune system in plants [23].

The GO annotation retrieved a total of 58,500 terms for the Biological Process (BP) category, 61,467 terms for the Molecular Function (MF) category and 62,795 terms for the Cellular Component (CC) category. In addition, a total of 62,077 transcripts were annotated in the Kyoto Encyclopedia Gene and Genomes (KEGG) database.

### Digital gene expression analysis

**Fig. 1:**
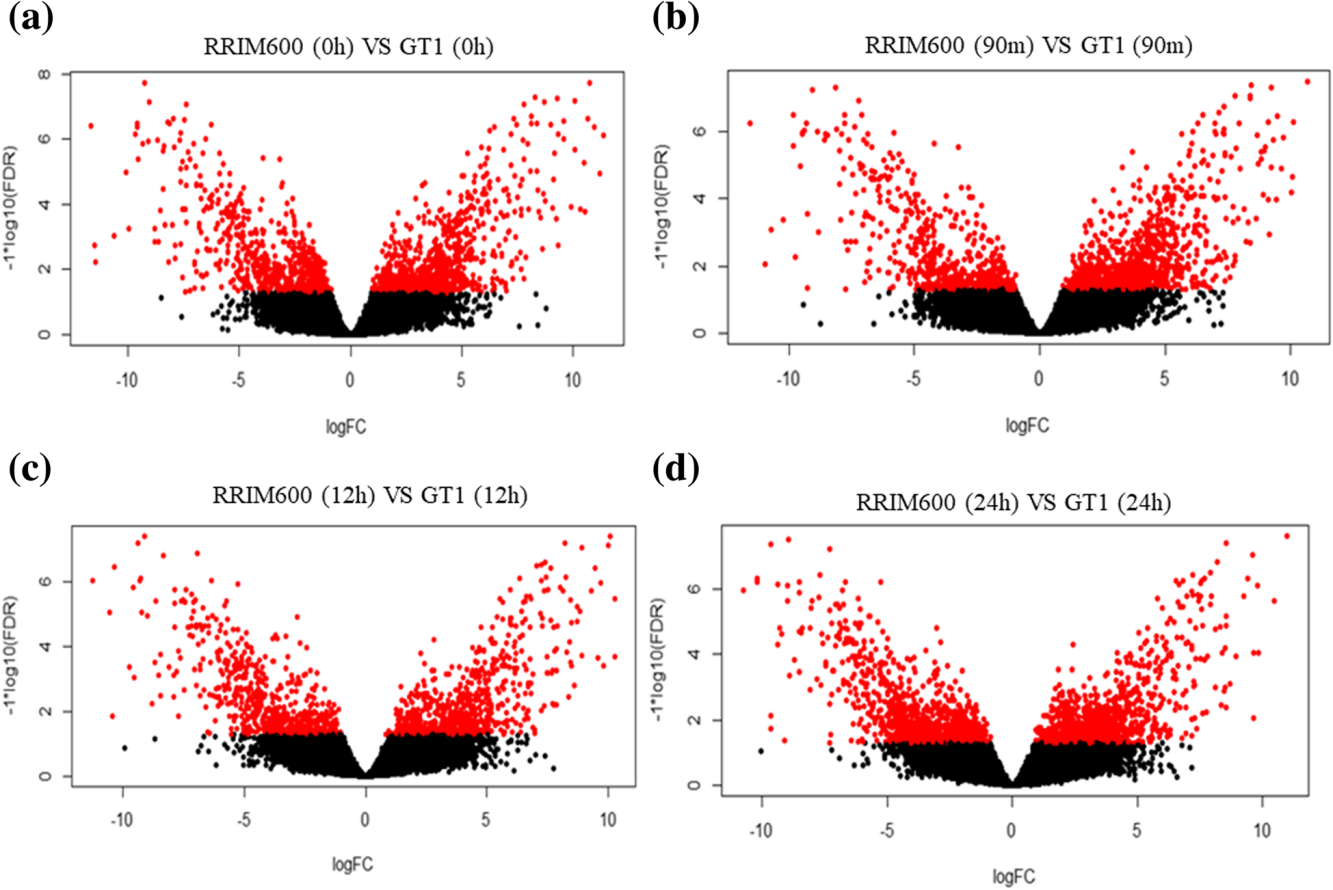
Volcano plot of the pairwise comparison between RRIM600 and GT1 for each time series

**Fig. 2:**
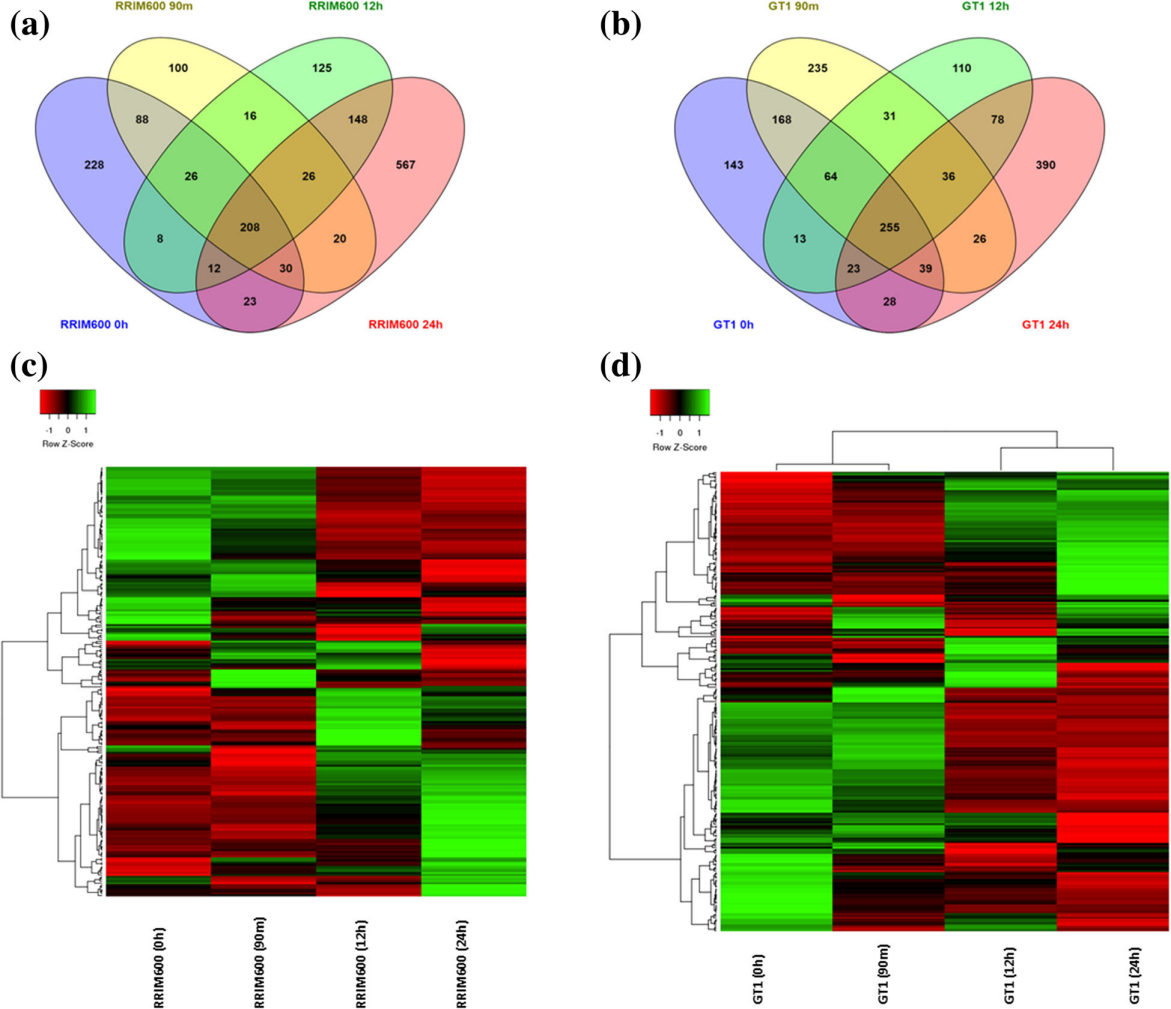
Expression profiles of the RRIM600 and GT1 genotypes. (**a**) Venn diagram representing the upregulated genes identified in RRIM600 throughout the chilling treatment. (**b**) Venn diagram representing the upregulated genes identified in GT1 throughout the chilling treatment. (**c**) Hierarchical clustering of the 208 common overexpressed genes in RRIM600. (**d**) Hierarchical clustering of the 255 common overexpressed genes in GT1

To compare the chilling stress response strategy between the rubber tree genotypes, we performed a pairwise comparison for each time series between RRIM600 and GT1. Prior to exposure to cold stress (0 h), 624 genes were upregulated in RRIM600, while 732 genes were downregulated. After 90 m of cold-stress exposure, 514 genes were upregulated and 854 genes were downregulated in RRIM600. Moreover, a total of 569 genes and 1034 genes were upregulated in RRIM600 after 12 h and 24 h of cold-stress exposure, respectively. In contrast, a total of 610 and 875 genes were downregulated in RRIM600 after 12 h and 24 h of cold treatment, respectively (Figs. 1 and 2, Additional file 3: Table S2).

Since the DEG analysis was performed by comparing the RRIM600 genotype to the GT1 genotype for each time point of cold treatment, we compared the up- and downregulated genes previously identified across all time points in order to identify genes that were commonly or exclusively upregulated in the relative comparisons at each treatment time. We detected a total of 229 genes that were exclusively upregulated at 0 h (Fig. 2a). After 90 min, 12 h and 24 h, we identified 100, 125 and 567 exclusively upregulated genes, respectively. Moreover, a total of 208 RRIM600 genes were commonly upregulated within the entire series (Fig. 2a and c, Additional file 3: Table S2).

A total of 143 downregulated genes were exclusively upregulated at 0 h, and 235, 110 and 390 genes were exclusively downregulated after 90 m, 12 h and 24 h, respectively (Fig. 2b). Furthermore, a total of 255 genes were commonly downregulated across the time series (Fig. 2b and d, Additional file 3: Table S2).

In addition, to clarify the strategy used to cope with cold tolerance, we performed DEG analysis within each genotype per time series. Among the three comparisons (0 X 90 m, 90 m X 12 h and 12 h X 24 h) in each genotype, we observed a significant change in the DEG number when we compared the DEGs at 90 m to those at 12 h. In RRIM600, we identified 48, 2188 and 53 upregulated genes and 3, 1372 and 9 downregulated genes at 90 m, 12 h and 24 h, respectively (Additional file 4: Table S3). In GT1, we detected a total of 21 up- and 1 downregulated genes at 90 m relative to 0 h. In addition, at 12 h and 24 h, 1075 and 17 genes were upregulated, respectively, and 531 and 4 genes were downregulated, respectively (Additional file 4: Table S3).

### Protein domain homology among DEGs

Prior to cold stress, we detected four genes with the Apetala 2 (AP2) domain that were upregulated in RRIM600. The AP2 genes show high similarity to ERF119, which may be involved in the regulation of gene expression by affecting stress factors and components of stress signal transduction pathways.

We also identified five upregulated genes containing the VQ motif in RRIM600 at 12 h (Table 2). This domain is a plant-specific domain characteristic of a class of proteins that regulates diverse developmental processes, including responses to biotic and abiotic stresses, seed development, and photomorphogenesis [24].

**Table 2:** The 10 most representative Pfam protein domain annotations for DEGs

Genotype	Treatment	Number of Genes	Pfam Domain
RRIM600 upregulated	0 h	13	Protein tyrosine kinase
		11	NB-ARC domain
		8	Protein kinase domain
		7	Plant protein of unknown function
		6	Cytochrome P450
		6	Leucine rich repeat N-terminal domain
		6	PPR repeat
		6	RNA recognition motif
		5	Ribosomal protein
		4	ADP-ribosylation factor family
		4	AP2 domain
		4	D-mannose binding lectin
		4	KH domain
		4	Leucine rich repeat
		4	Ribosomal protein L11, N-terminal domain
		4	Ribosomal protein S8
RRIM600 downregulated	0 h	16	Leucine rich repeat N-terminal domain
		12	Cytochrome P450
		12	Protein kinase domain
		11	NB-ARC domain
		10	PMR5 N-terminal domain
		8	Ankyrin repeats (3 copies)
		8	Glycosyl transferase family 8
		7	Microtubule binding
		6	IQ calmodulin-binding motif
		6	Legume lectin domain
		6	Multicopper oxidase
		6	Zinc-binding RING-finger
		5	Myb-like DNA-binding domain
		5	Peptidase inhibitor I9
		5	Short chain dehydrogenase
		5	TIR domain
RRIM600 upregulated	90 m	13	NB-ARC domain
		13	Protein tyrosine kinase
		9	Leucine rich repeat N-terminal domain
		8	Protein kinase domain
		6	D-mannose binding lectin
		5	Leucine rich repeat
		5	Plant protein of unknown function
		4	Cytochrome P450
		4	UDP-glucuronosyl and UDP-glucosyl transferase
		3	Ammonium Transporter Family
		3	CCAAT-binding transcription factor (CBF-B/NF-YA) subunit B
		3	Leucine rich repeat
		3	TIR domain
		3	Wall-associated receptor kinase C-terminal
		3	Wall-associated receptor kinase galacturonan-binding
RRIM600 downregulated	90 m	25	Protein kinase domain
		22	Leucine rich repeat N-terminal domain
		15	NB-ARC domain
		13	PMR5 N-terminal domain
		12	Cytochrome P450
		11	Zinc-binding RING-finger
		9	Ankyrin repeats (3 copies)
		9	Glycosyl transferase family 8
		8	IQ calmodulin-binding motif
		7	Microtubule binding
		7	Short chain dehydrogenase
RRIM600 upregulated	12 h	17	NB-ARC domain
		10	Protein tyrosine kinase
		9	Leucine rich repeat N-terminal domain
		7	Protein kinase domain
		5	F-box domain
		5	Plant protein of unknown function
		5	TIR domain
		5	UDP-glucuronosyl and UDP-glucosyl transfer
		5	VQ motif
		4	Cytochrome P450
		4	D-mannose binding lectin
		4	Leucine rich repeat
		4	PPR repeat
RRIM600 downregulated	12 h	25	Protein kinase domain
		22	Leucine rich repeat N-terminal domain
		15	NB-ARC domain
		13	PMR5 N-terminal domain
		12	Cytochrome P450
		11	Zinc-binding RING-finger
		9	Ankyrin repeats (3 copies)
		9	Glycosyl transferase family 8
		8	IQ calmodulin-binding motif
		7	Microtubule binding
		7	Short chain dehydrogenase
RRIM600 upregulated	24 h	17	NB-ARC domain
		15	UDP-glucuronosyl and UDP-glucosyl transferase
		14	Cytochrome P450
		14	PPR repeat
		12	Protein kinase domain
		11	Leucine rich repeat N-terminal domain
		11	Protein tyrosine kinase
		9	D-mannose binding lectin
		9	Short chain dehydrogenase
		8	AP2 domain
RRIM600 downregulated	24 h	23	Leucine rich repeat N-terminal domain
		21	Protein kinase domain
		20	NB-ARC domain
		11	TIR domain
		9	Protein tyrosine kinase
		8	Ankyrin repeats (3 copies)
		8	Cytochrome P450
		7	Microtubule binding
		7	PMR5 N-terminal domain
		6	Carbohydrate-binding protein of the ER
		6	Salt stress response/antifungal

After 24 h of cold treatment, the most abundant domain among the upregulated genes in RRIM600 was the NB-ARC domain, which is a signaling motif that is shared by plant resistance products and is a regulator of cell death in animals [25]. The next two most common domains were *UDP-glucuronosyl*/*UDP-glucosyl transferase*, which catalyzes the transfer of sugars to a wide range of acceptor molecules and regulates their activities [26], followed by *cytochrome P450* (CYP450). CYP450 catalyzes diverse reactions leading to the precursors of structural macromolecules, such as lignin and cutin, and is involved in the biosynthesis or catabolism of hormone and signaling molecules, such as antioxidants and defense compounds [27] (Table 2). Furthermore, eight upregulated genes containing the AP2 domain were identified in RRIM600 at 24 h.

LRR N-terminal domains were the most abundant type of domain that was downregulated in RRIM600 at 24 h, followed by protein kinase domains and NB-ARC domains. The LRR N-terminal domain is involved in a number of biological processes, including cell adhesion, signal transduction, immune responses, apoptosis and disease resistance [28]. Moreover, six downregulated genes with a salt stress response/antifungal domain were identified in RRIM600 at 24 h (Table 2).

Interestingly, we identified two upregulated genes containing the *cold shock domain-containing protein 3* (CSP3) domain in RRIM600 at 0 h. CSP3 shares a cold shock domain with bacterial CSPs and is involved in the acquisition of freezing tolerance in plants. In *Arabidopsis*, overexpression of genes containing CSP3s in transgenic plants confers enhanced freezing tolerance [29].

The pentatricopeptide repeat (PPR) superfamily is one of the largest gene families in plants. For example, more than 400 members of this group have been identified in both rice and Arabidopsis [30]. Most PPR proteins are targeted to the chloroplast and mitochondria and are involved in many functions. They play important roles in responses to developmental and environmental stresses. Additionally, a set of PPR genes has been reported to be involved in abiotic stress response regulation in Arabidopsis through ROS homeostasis or ABA signaling [50]. In this study, we observed that the number of upregulated PPR genes increased in RRIM600 after 24 h of cold treatment. Prior to cold stress, RRIM600 showed six upregulated genes and three downregulated genes. However, after 24 h of chilling stress, RRIM600 showed 14 upregulated PPR genes and seven downregulated PPR genes (Additional file 5: Table S4).

### DEG GO enrichment

To identify enriched GO terms, we performed a GO enrichment analysis with the up- and downregulated genes identified in RRIM600 relative to GT1 for each time point (RRIM600 0 h X GT1 0 h; RRIM600 90 m X GT1 90 m; RRIM600 12 h X GT1 12 h; RRIM600 24 h X GT1 24 h) (Additional file 4: Table S3). Among all terms identified for all upregulated genes, a total of 32 nonredundant BP terms were identified across the time series in RRIM600. Furthermore, we detected a total of 20 and 31 distinctive terms in the CC and MF categories, respectively. RRIM600 presented a total of 102 nonredundant terms in the BP category in the set of downregulated genes. The CC and MF categories exhibited a total of 37 and 44 unique terms, respectively.

We observed enriched BP terms related to defense, such as the defense response (GO:0006952), cellular defense response (GO:0006968) and innate immune response (GO:0045087), in the upregulated genes prior to cold stress (0 h). Additionally, we observed a substantial increase in the number of sequences related to the defense response category. Before cold treatment, RRIM600 contained 71 upregulated genes in this category. After 24 h, we observed a total of 97 genes associated with defense responses (Additional file 5: Table S4). Interestingly, we also observed that the downregulated gene set in RRIM600 was enriched for the lignin biosynthetic process (GO:0009809) and lignin metabolic process (GO:0009808) at 90 min. Across all time points, the downregulated gene set in RRIM600 was enriched for categories such as cell wall (GO:0042546), plant-type cell wall biogenesis (GO:0009832), plant-type cell wall organization or biogenesis (GO:0071669) and cell wall biogenesis (GO:0042546) (Additional file 5: Table S4).

After 24 h at 10 °C, the respiratory chain category was enriched in the upregulated genes in RRIM600 (GO:0070469), whereas the downregulated genes exhibited enriched terms such as thylakoid part (GO:0044436), photosystem II (GO:0009523) and photosystem II oxygen evolving complex (GO:0009654) (Additional file 5: Table S4).

For the MF category, the number of downregulated genes annotated with cellulose synthase activity (GO:0016759) increased from 10 to 14 between 0 h and 90 min in RRIM600. In contrast, at 24 h of cold stress, the number of cellulose synthase genes decreased to 9. Interestingly, the upregulated gene set of RRIM600 did not show any enriched categories related to cellulose synthase or the lignification process during cold treatment.

In RRIM600, the categories purine ribonucleotide binding (GO:0032555), purine nucleotide binding (GO:0017076), purine nucleoside binding (GO:0001883) and purine ribonucleoside binding (GO:0032550) were enriched from 90 min to 24 h of chilling treatment. Furthermore, the number of upregulated genes related to these GO categories increased during cold treatment (Additional file 5: Table S4).

### Putative molecular marker detection

The comprehensive transcriptome was evaluated by searching for microsatellites (simple sequence repeats – SSRs) and SNPs. For the SSR analysis, a total of 21,237 transcripts contained at least one SSR motif, with 4570 transcripts with more than 1 SSR per sequence. The SSR frequency in this transcriptome was 1 SSR per 7.2 kb (Additional file 6: Table S5).

Among the total putative SSRs detected, 16,621 (61%) were classified as dinucleotides, followed by 9336 (34%) tri-, 634 (2%) tetra-, 283 (1%) penta- and 237 (1%) hexanucleotides. Among the dinucleotide SSRs, the most abundant motif (12,075, 72.65%) was AG/TC, followed by the AT/TA, AC/TG and GC/CG motifs, with abundances of 2939 (17.68%), 1560 (9.38%) and 47 (0.2%), respectively (Additional file 6: Table S5).

The upregulated genes detected in the GT1 and RRIM600 genotypes were merged to identify putative SSRs. A total of 1034 dinucleotide SSRs were identified, while 629 tri-,18 tetra-,19 penta- and 23 hexanucleotide SSRs were identified (Additional file 6: Table S5).

SNP calling was performed for each genotype using Freebayes. A total of 202,949 putative SNPs were detected in GT1. Transition (Ts) SNPs were more abundant than transversion (Tv) SNPs, resulting in a Ts/Tv ratio of 1.46. Among the Ts variations, A↔G was the most abundant, with 61,111 putative SNPs, while A↔T was the most abundant variation for the Tv SNPs, with 24,613 markers (Additional file 7: Table S6). The SNP frequency for GT1 was 1 SNP per 967 bp.

For the RRIM600 genotype, a total of 156,354 putative SNPs were detected, and the Ts/Tv ratio was 1.53. As observed in GT1, A↔G was the most abundant variation, with 48,196 SNPs. The most frequent variation among the Tvs was A↔T, with 18,525 putative SNPs. The SNP frequency was 1 SNP per 1255 kb (Additional file 7: Table S6).

A total of 94,962 SNPs were common between GT1 and RRIM600, and the overall SNP frequency was 1 SNP per 742 bp. Among the DEGs, we identified 20,203 and 14,998 SNPs in GT1 and RRIM600, respectively. Among the SNPs identified in DEGs, 12,509 SNPs were exclusive to GT1, and 7484 SNPs were exclusive to RRIM600.

### qRT-PCR validation

To validate the DEG analysis, a total of 20 genes were randomly selected. All primer pairs were initially tested via PCR using genomic DNA as a template to verify the amplification product. From the 20 primer pairs, 14 were successfully amplified and used for qRT-PCR.

**Fig. 3:**
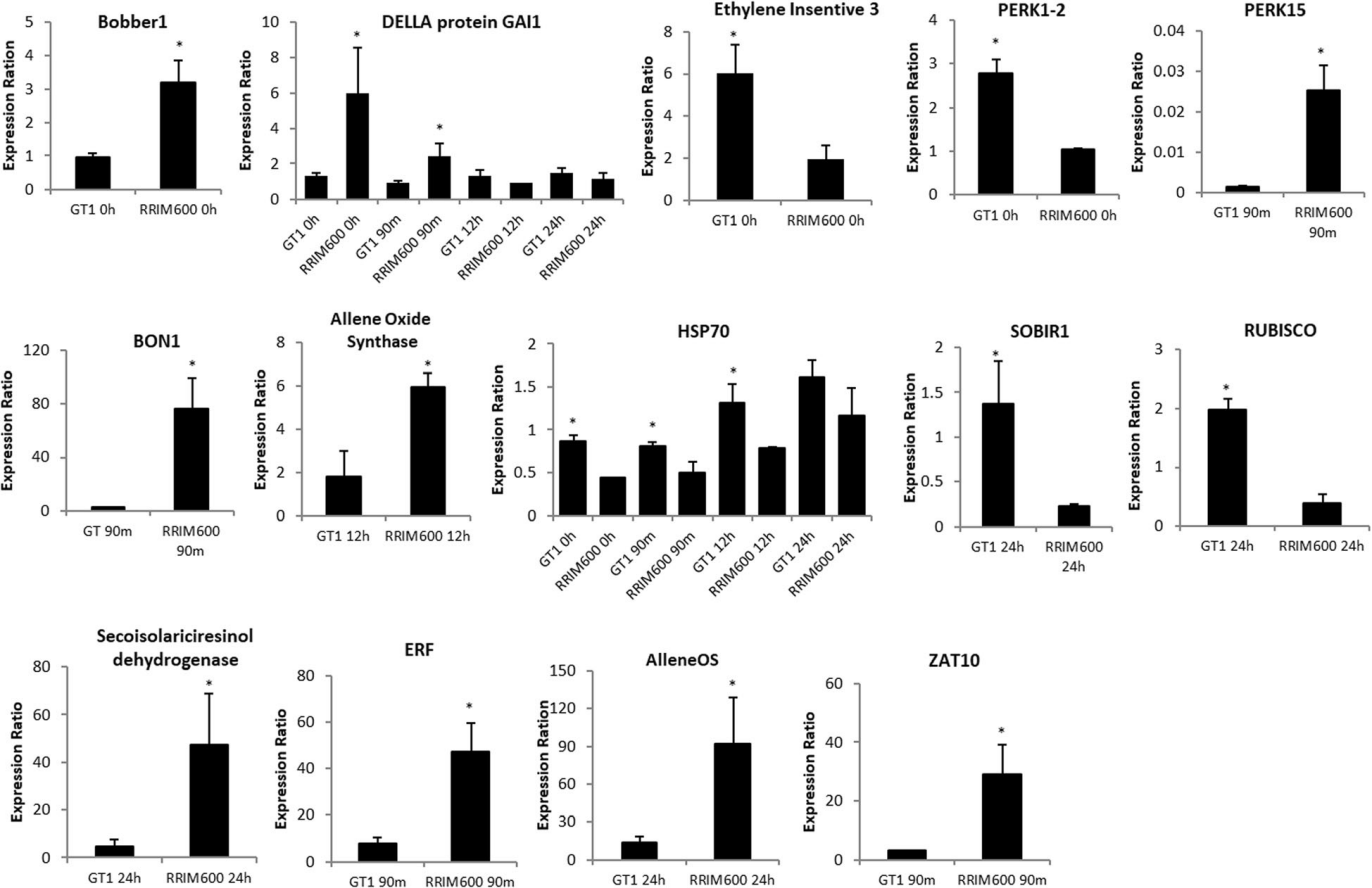
qPCR expression analysis of the 14 genes identified in the in silico DEG analysis. The expression values represent the mean (*n* = 3 or n = 2) ± SEM. The bars indicate the SEM values, and significant differences (p < 0.05) are indicated with asterisks

Among the 14 genes tested (Fig. 3), 11 were differentially expressed between RRIM600 and GT1 and confirmed by the in silico analysis. The gene encoding the DELLA protein GAI1, a repressor of the GA signaling pathway [34] (PASA_cluster_35787), was detected in the in silico analysis as upregulated in RRIM600 across all time points; however, the qPCR results revealed that this gene was upregulated at only 0 h and 90 min of cold stress. For the HSP70 gene (PASA_cluster_30195), which was also identified as downregulated across all time points in RRIM600 in the in silico analysis, qPCR confirmed that the HSP70 gene was downregulated at the 0 h, 90 min and 12 h time points. After 24 h, HSP70 levels were lower in RRIM600 than in GT1, but this difference was not significant (Fig. 3).

The only gene with qRT-PCR results that were not in accordance with the in silico analysis was the protein *ETHYLENE INSENSITIVE 3* (EIN3) (PASA_cluster_52015). The in silico analysis showed EIN3 was upregulated in RRIM600; however, the qRT-PCR results showed that this gene was significantly downregulated in RRIM600 (Fig. 3).

### Alternative splicing detection

AS is an important mechanism involved in gene regulation that may regulate many physiological processes in plants, including responses to abiotic stresses such as cold stress [56]. It has been estimated that 60% of the genes in Arabidopsis are subject to AS [57]. Furthermore, studies in soybean and maize predicted that 52% [31] and 40% [32] of all genes are subject to AS events.

**Fig. 4:**
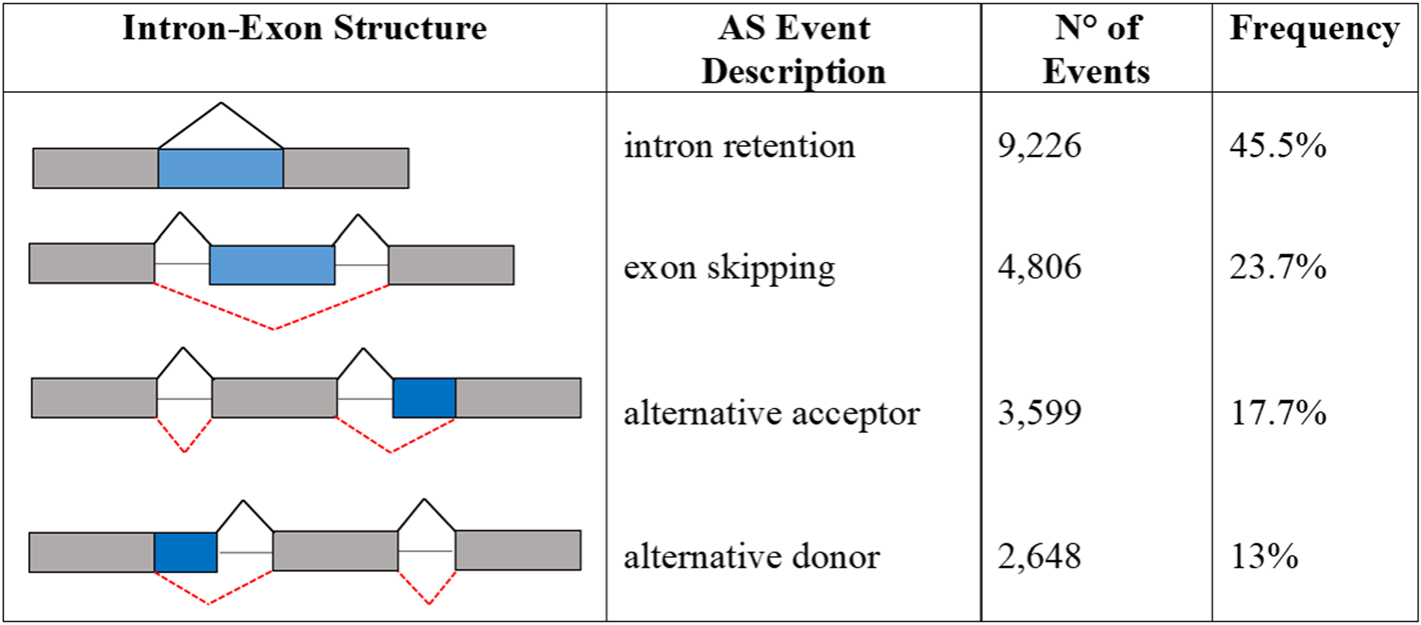
Summary of AS detection in the comprehensive transcriptome

Due to the importance of AS, the reference transcriptome obtained in this study was used to detect AS events. A minimum depth of 10 reads was used as the threshold to identify isoforms. A total of 20,279 AS events were identified. Intron retention (IR), accounting for a total of 9226 events (45.5%), represented the major type of AS event, followed by exon skipping (ES), alternative acceptor (AltA) and alternative donor (AltD) events, which accounted for 4806 (23.7%), 3599 (17.7%) and 2648 (13%) events, respectively (Fig. 4).

Although ES accounts for the majority of AS events in humans, it has been reported that IR events are the most abundant type of AS in plants [33].

## Discussion

Abiotic stress is caused by environmental conditions such as cold and drought, which consequently affect optimum growth and yields. Environmental factors may represent as much as 70% of all factors that influence crop production [35]. The inhibition of growth is one of the earliest responses to abiotic stress. The metabolism of lipids and sugar and photosynthesis are affected gradually as the stress becomes more severe and/or prolonged. The plant response to abiotic stresses is complex and involves interactions and crosstalk with many molecular pathways [35]. Therefore, one stress tolerance mechanism could be defined as the ability to detect stress factors and respond to the factors appropriately and efficiently [36].

### Reactive oxygen species (ROS)

ROS are continuously produced at basal levels under favorable conditions. Organisms exhibit antioxidant mechanisms that scavenge ROS to maintain an appropriate ROS balance [37].

In addition, different types of stress factors, such as drought, pathogen infection and extreme temperatures, disturb the balance between ROS generation and ROS scavenging, causing oxidative damage to membranes, proteins, RNA and DNA [38].

The survival of plants therefore depends on many important factors, such as changes in growth conditions, the severity and duration of stress conditions and the capacity of the plants to quickly adapt to changing energy equations [39]. Under stressful conditions, plant redox homeostasis is maintained by both antioxidant enzymes, such as pH-dependent peroxidases (POXs), superoxide dismutase (SOD), ascorbate peroxidase (APX), guaiacol peroxidase (GPX), glutathione-S-transferase (GST), and catalase (CAT), and nonenzymatic compounds, such as ascorbic acid (AA), reduced glutathione (GSH), α-tocopherol, carotenoids, phenolics, flavonoids, and proline [39, 40].

**Fig. 5:**
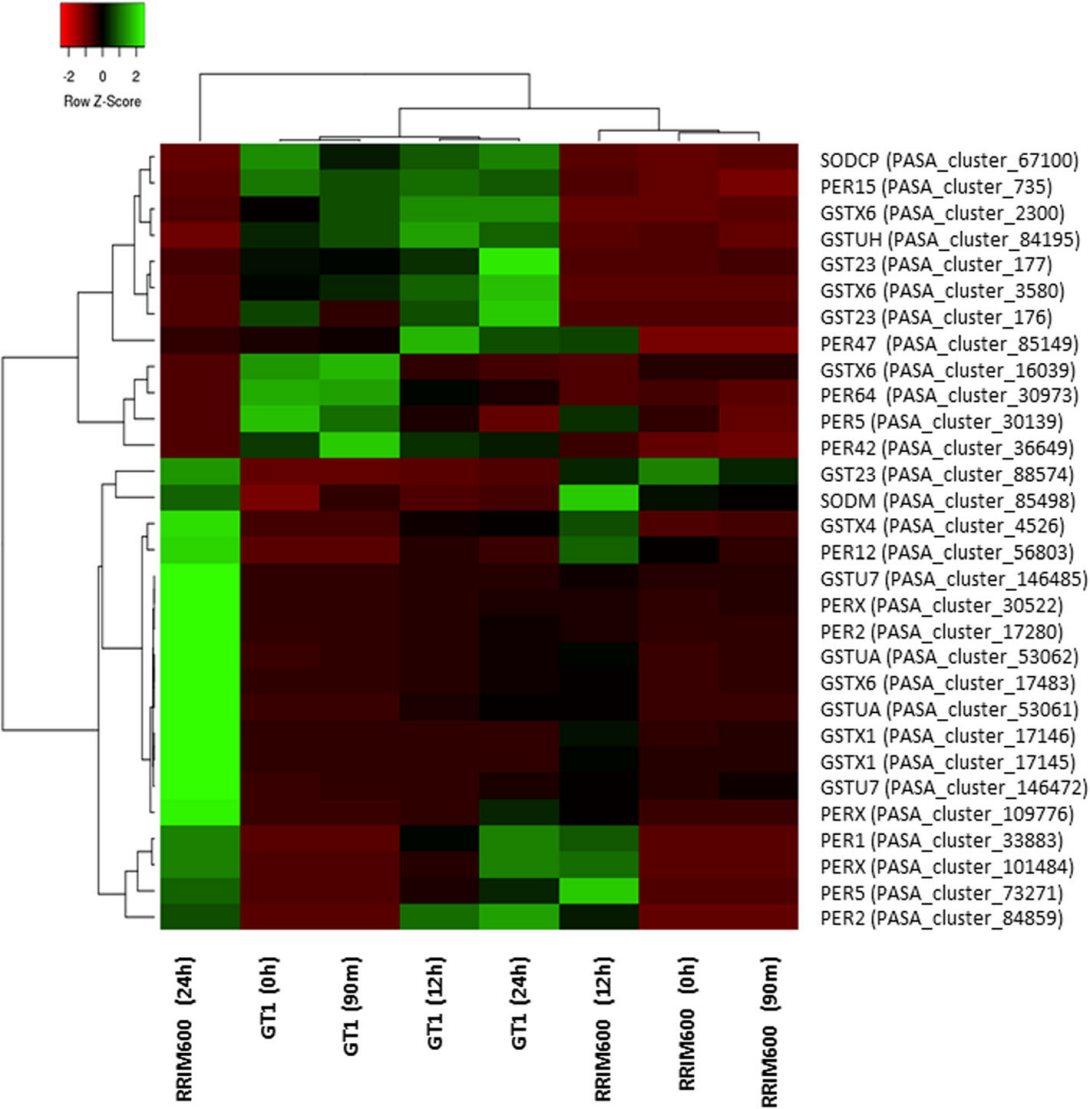
Heat map of ROS scavenging for each genotype in each time series

The pairwise comparison between RRIM600 and GT1 samples collected before exposure to cold stress revealed that RRIM600 had one upregulated and two downregulated genes with similarity to peroxidase. After 90 m, RRIM600 exhibited a total of four downregulated genes with best BLAST hits to peroxidase. However, after 24 h of treatment, only one upregulated gene with probable peroxidase activity was identified among the three genes (Fig. 5, Additional file 3: Table S2).

In addition, the gene expression within each genotype revealed that there were seven genes with peroxidase activity in RRIM600 upregulated after 12 h (RRIM600 90 m X RRIM600 12 h) of cold stress, while GT1 showed only three upregulated genes with peroxidase activity (GT1 90 m X GT1 12 h). The three upregulated genes identified in GT1 were also upregulated in RRIM600 (Fig. 5, Additional file 4: Table S3), showing that RRIM600 recruited more peroxidase genes than GT1 during cold treatment, even though only one gene (*cationic peroxidase 2*; PASA_cluster_17280) among of these was identified as differentially expressed between the genotypes (RRIM600 X GT1). We observed that the *cationic peroxidase 2* (PASA_cluster_17280) gene was upregulated in RRIM600 after 12 h (RRIM600 90 m X RRIM600 12 h) and between RRIM600 and GT1 after 24 h (RRIM600 24 h X GT1 24 h) (Fig. 5, Additional file 3: Table S2, Additional file 4: Table S3). In addition, the DGE analysis revealed that this gene showed a high transcript level change after 24 h of treatment.. Deng et al. (2018) also observed several types of peroxidases that exhibited sharp changes in expression between cold-tolerant and cold-susceptible clones during cold stress treatment [20].

Putative GST genes were identified across the time series. Prior to cold stress treatment, one GST gene was upregulated and three GST genes were downregulated in RRIM600 (Fig. 5a). After 90 min, we detected five genes with high similarity to GST genes. In RRIM600, four of these genes were downregulated, and one of these genes was upregulated. At the subsequent time point, we observed an increase in upregulated GST genes in RRIM600. After 24 h, RRIM600 had nine upregulated and four downregulated putative *GST* genes (Fig. 5, Additional file 4: Table S3).

Furthermore, we identified two upregulated putative *thioredoxin H1* (*TRXh1*) genes in RRIM600 after 24 h of cold stress. In *Oryza sativa*, the *TRXh1* gene is involved in stress responses by regulating the balance of ROS in the rice apoplast. *TRXh1* plays an important role in redox state regulation and stress responses [41] (Additional file 3: Table S2).

It has been reported that ROS scavenging efficiency may be one of the fundamental elements that can distinguish cold-susceptible from cold-tolerant plants in species such as rubber tree [20], *Camellia sinensis* [42], and Arabidopsis [43].

According to our findings, ROS scavenging efficiency could also be associated with the coping strategies of RRIM600 and GT1 under cold stress. Although peroxidases are known to play a role in ROS scavenging, we did not find significant differences in terms of up- or downregulated peroxidase genes across the time series in RRIM600 relative to GT1. However, RRIM600 showed a significant increase in the number of upregulated *GST* genes, which, combined with the other ROS scavenging genes identified in this study, could indicate that *GST* genes play an important role in the ROS scavenging efficiency of this clone.

### Signal transduction

In general, the perception of stress is followed by the generation of second messengers (inositol phosphates and ROS), which modulate the influx of cytosolic Ca^2+^, initiate a protein phosphorylation cascade and activate targeted proteins in cellular protection or transcription factors controlling stress-regulated genes [44].

This increase in cytosolic Ca^2+^ is considered an important messenger for signal transduction and therefore cold acclimation. In alfalfa and Arabidopsis, a positive correlation between the cold-induced cytosolic Ca^2+^ increase and the accumulation of cold-induced transcripts has been observed [45, 46]. Ca^2+^ from the cytosol can be detected by Ca^2+^ sensors, such as *calmodulin* (CaM), *CaM-like proteins* (CML), *CaM domain-containing protein kinases* (CDPKs), *calcineurin B-like proteins* (CBLs) and CBL-interacting protein kinases (CIPKs) [46].

**Fig. 6:**
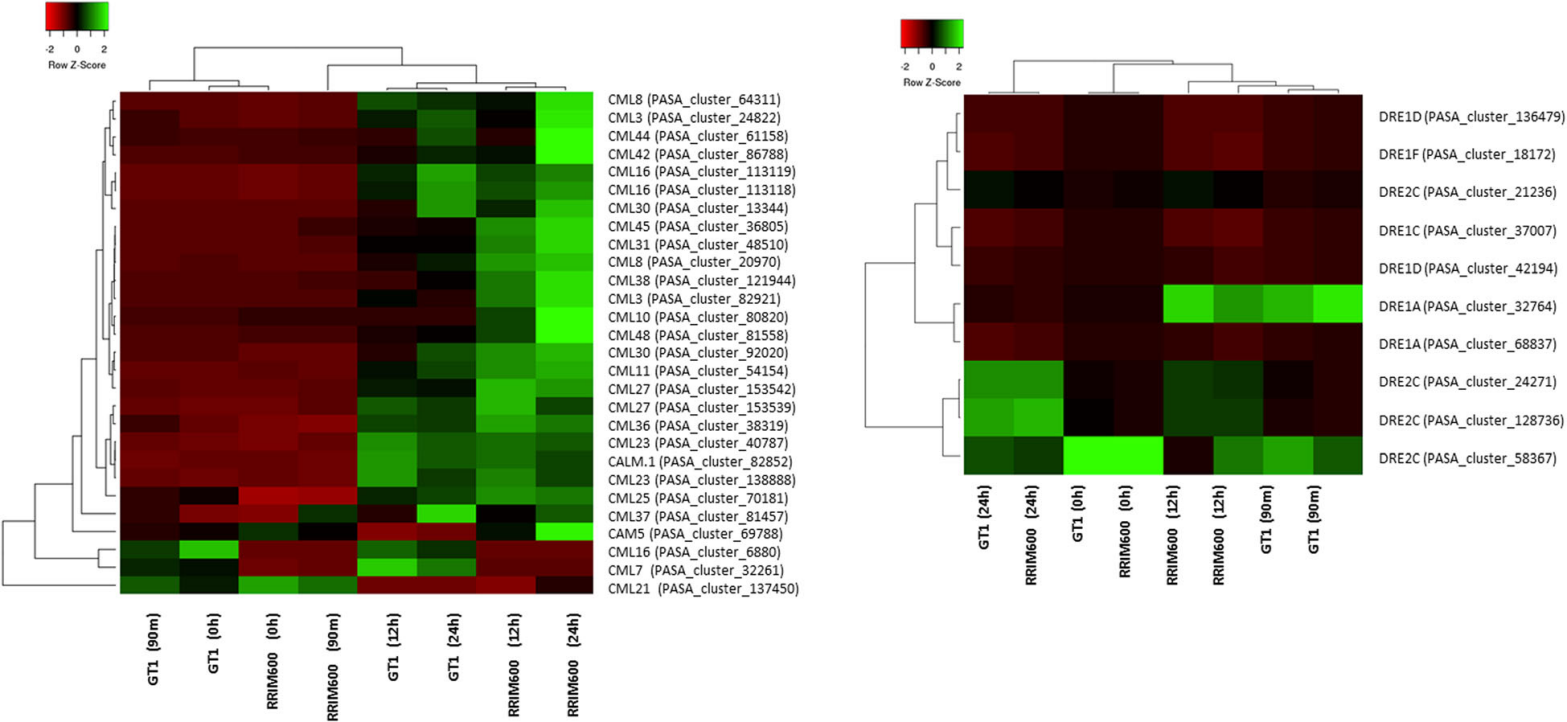
Heat map of up- and donwregulated signal transduction genes identified in each time series. (**a**) CAM and CMLs. (**b**) CAMTAs and DREBs

The comparison between RRIM600 X GT1 detected one CML45 upregulated in RRIM600 after 90 m of cold stress (RRIM600 90 m X GT1 90 m). In addition, one *CML10* and one *CML38* gene were upregulated in RRIM600 after 12 h (RRIM600 12 h X GT1 12 h). After 24 h, one *CML48* and one *CAM*5 gene were upregulated in RRIM600, while the CML10 expression level was maintained (RRIM600 24 h X GT1 24 h) (Fig. 5b Additional file 3: Table S2). Regarding the downregulated CML genes in the comparison between RRIM600 and GT1, we detected one *CML16* gene that was downregulated in RRIM600 prior to cold stress and after 12 h of treatment and one *CML7* that was downregulated in RRIM600 after 12 h of cold stress (Fig. 6a, Additional file 3: Table S2).

The gene expression analysis within each genotype revealed that a total of 23 CML genes were upregulated in RRIM600, i.e., one gene at 90 m (RRIM600 0 h X RRIM600 90 m), 21 genes at 12 h (RRIM600 90 m X RRIM600 12 h) and one gene at 24 h (RRIM600 12 h X RRIM600 24 h). In GT1, CML genes were upregulated only after 12 h of cold stress (GT1 90 m X GT1 12 h). Among the 15 upregulated genes identified in GT1, 13 were commonly upregulated in RRIM600 (Additional file 4: Table S3). Interestingly, three upregulated genes that were exclusively identified in RRIM600 (RRIM600 90 m X RRIM600 12 h) after 12 h of cold stress were also upregulated RRIM600 relative to GT1 (RRIM600 X GT1) (CML38, CML45 and CML10) and had high transcript levels (Fig. 6a, Additional file 4: Table S3), which could be indicative of their importance in signal transduction under cold stress.

Although the comparison between RRIM600 and GT1 did not reveal differentially expressed calmodulin binding transcription activator (*CAMTA*) family genes, we identified one *CAMTA2* gene that was upregulated after 12 h relative to 90 m of cold stress in RRIM600 and in GT1 (RRIM600 90 m X RRIM600 12 h; GT1 90 m X GT1 12 h). In addition, in both genotypes, one *CAMTA3* gene was downregulated after 12 h (Fig. 6b, Additional file 4: Table S3). In Arabidopsis, *CAMTA2* and *CAMTA3* are positive regulators of *C-REPEAT 2/DRE BINDING FACTOR 1C* (*CBF2/DREB1C*) gene expression and are involved in cold tolerance [47].

Under cold stress, the signaling cascade can activate CBF transcription factors, which can bind to cis-elements in the promoters of cold-regulated (COR) genes and activate their expression [43]. These CBF genes have been reported to enhance freezing tolerance in several species, such as Arabidopsis [43]. In this study, we detected one upregulated CBF3/DREB1A in RRIM600 after 12 h of cold stress (RRIM600 90 m X RRIM600 12 h). In addition, eight DREB genes (one *DREB1A*, one *DREB1C*, two *DREB1Ds*, one *DREB1F* and three *DREB2*As) were upregulated in RRIM600 at 12 h (RRIM600 90 m X RRIM600 12 h), while GT1 (GT1 90 m X GT1 12 h) upregulated four *DREB2C* genes (Fig. 6b, Additional file 4: Table S3). Similar to other species, *CBF/DRE* genes are involved in cold tolerance in rubber trees, as indicated by analysis of cold-susceptible versus cold-tolerant genotypes [20]. Although *CBF/DRE* genes are important for activating COR genes, these transcription factors were not differentially expressed in RRIM600 relative to GT1. These findings suggest that the strategy adopted to cope with cold stress does not differ with respect to the CBF regulon, as was observed in *Alternanthera philoxeroides* [48].

**Fig. 7:**
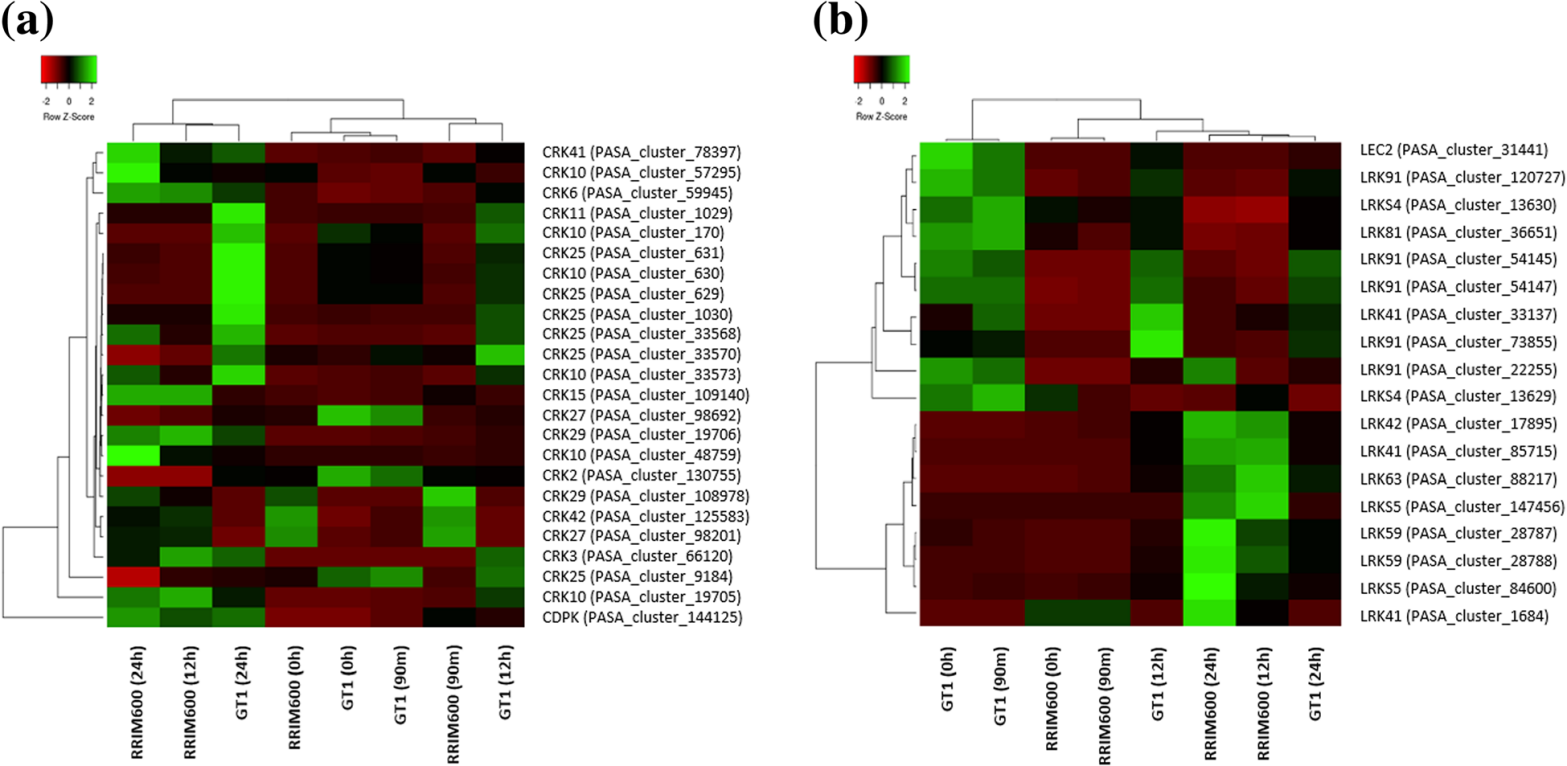
Heat map of up- and downregulated protein kinase signal transduction genes. (**a**) CRKs and (**b**) LRKs identified in each time series

Cysteine-rich receptor-like kinases (CRKs) can significantly affect plant development and stress responses [49]. It has been suggested that CRK transcript levels are elevated in response to salicylic acid (SA), pathogens and drought. Additionally, CRKs are involved in mediating the effects of ROS [50]. Before chilling stress, the comparison between RRIM600 to GT1 revealed three up- and four downregulated CRK genes. However, after 24 h of cold treatment, RRIM600 exhibited four up- and five downregulated CRK genes; of these, *CRK29* (PASA_cluster_108978) and *CRK27* (PASA_cluster_98201) were also upregulated prior to cold stress. Interestingly, all of the downregulated genes identified in RRIM600 after 24 h differed from the previously upregulated CRK genes (Fig. 7a, Additional file 3: Table S2). In addition, the comparison between the time series within the genotype RRIM600 revealed one CRK10 gene upregulated after 90 m (RRIM600 0 h X RRIM600 90 m) and 10 upregulated CRK genes and one downregulated CRK gene after 12 h of cold stress (RRIM600 90 m X RRIM600 12 h), with one *CRK10* gene (PASA_cluster_48759) also upregulated after 12 h and 24 h of cold stress when RRIM600 was compared to GT1 (RRIM600 12 h X GT1 12 h; RRIM600 24 h X GT1 24 h) (Fig. X, Additional file 3: Table S2 and Additional file 4: Table S3). Although we did not observe large differences in the number of up- and downregulated genes in RRIM600, the two CRK genes (PASA_cluster_108978 and PASA_cluster_98201) that were upregulated prior to cold stress and were maintained after 24 h could play an important role in cold stress signaling.

The recognition of abiotic stress signals initiates specialized signaling pathways in which phosphatases and protein kinases, such as CDPKs, CBLs, CIPKs and receptor-like kinases (RLKs), including LRR_RLK, MRLK and Lectin RLK (LecRLK) [51], which are LecRLK plant-specific proteins, are key components [52].

In this study, we detected a significant increase in the number of upregulated LecRLK genes in RRIM600 relative to GT1 after 24 h of cold treatment (RRIM600 24 h X GT1 24 h). Before the cold treatment, seven LecRLK genes were downregulated in RRIM600; however, after 24 h, we identified a total of eight LecRLK genes that were upregulated in RRIM600 (Fig. 6a, Additional file 3: Table S2). Interestingly, the analysis within each genotype revealed that six (PASA_cluster_28788, PASA_cluster_147456, PASA_cluster_28787, PASA_cluster_17895, PASA_cluster_84600, PASA_cluster_85715) of the eight LecRLK genes upregulated in RRIM600 24 h (RRIM600 24 h X GT1 24 h) were also upregulated in RRIM600 (RRIM600 90 m X RRIM600 12 h) and GT1 (GT1 90 m X GT1 12 h) after 12 h of cold stress (Fig. 7b, Additional file 3: Table S2 and Additional file 4: Table S3). LecRLK genes were previously reported to enhance resistance to pathogen infection in tobacco [53] and Arabidopsis [54], to play a role in abiotic stress signal transduction [55] and to upregulate stress-responsive genes [56–58]. Both clones upregulated the same six LecRLK genes: one *LRK41* gene, one *LRK42* gene, two *LRKS5* genes, and two *LRK59* genes. Therefore, the transcript levels of these genes were also differentially expressed in RRIM600 relative to GT1 (RRIM600 24 h X GT1 24 h) (Fig. 7b, Additional file 3: Table S2 and Additional file 4: Table S3). These overexpression of these genes in RRIM600 compared to GT1 could indicate a greater recognition of cold stress signals in the former compared to the latter.

### Photosynthesis activity and stomata closure

Cold stress can cause an imbalance between light utilization and energy dissipation through metabolic activity. Under this stress, an excess of photosystem II (PSII) excitation pressure exists, which can be reversible through the dissipation of excess absorbed energy or the irreversible inactivation of PSII and consequent inhibition of the photosynthetic capacity [59]. An imbalance in PSII caused by cold stress might generate ROS, which can then damage the photosynthetic apparatus and the whole cell [60]. Therefore, tolerance to cold-induced photoinhibition can be considered a mechanism for cold tolerance. Additionally, RuBisCO plays a central role in CO_2_ assimilation and photosynthesis efficiency. Crops that are acclimated to cold, such as winter wheat and rye, adjust their RuBisCO content and are able to maintain a high CO2 assimilation rate [61].

Low temperature can also affect the enzymes and ion channels responsible for maintaining the guard cell osmotic potential [62]. Due to the reduction in the photosynthetic capacity caused by cold stress, there is an increase in internal CO_2_ in the substomatal cavity, which reduces the stomatal aperture. Stomatal closure also favors the formation of ROS due to the decay of intracellular CO_2_ and increase in photorespiration [63].

In this study, GO enrichment analysis revealed categories related to photosynthesis were enriched for downregulated genes in RRIM600 relative to GT1 after 24 h of cold stress (RRIM600 24 h X GT1 24 h). We observed enriched GO categories associated with photosystem II (GO:0009523), photosystem (GO:0009521), photosynthetic membrane (GO:0034357), chlorophyll biosynthetic process (GO:0015995), and photosynthesis (GO:0015979) among the set of downregulated genes in RRIM600 after 24 h (RRIM600 24 h X GT1 24 h) (Additional file 4: Table S3). Additionally, the qPCR validation corroborated the in silico DEG analysis, in which one RuBisCO gene was found to be downregulated in RRIM600.

The pairwise comparison between RRIM600 and GT1 revealed that one abscisic acid (ABA) receptor *PYL4* gene was upregulated in RRIM600 at 0 h, 90 min and 24 h of chilling stress. Additionally, after 24 h of cold stress, RRIM600 upregulated two additional abscisic acid receptor *PYL4* genes and one ABA receptor *PYR1* gene (Additional file 3: Table S2). *PYR/PYL* ABA receptor genes are involved in ABA-mediated responses and play a major role in basal ABA signaling for vegetative and reproductive growth, modulation of the stomatal aperture and the transcriptional response to the hormone [64]. Recent studies have demonstrated that overexpression of the PYR1 gene in poplar significantly reduces the content of H_2_O_2_ and significantly contributes to cold tolerance [65].

Furthermore, we identified one upregulated gene with high similarity to the *HT1* gene in RRIM600 after 12 and 24 h of chilling stress. In Arabidopsis, the *HT1 serine/threonine kinase* gene is reported to be involved in the control of stomatal movement in response to CO_2_. Additionally, genes with high similarity to *hexokinase-1* and *PtdIns3P 5-kinase* (*PI3P5K*) were upregulated in RRIM600 at 24 h of treatment. These genes have also been reported to be related to stomatal closure via ABA [66] (Additional file 3: Table S2).

In Arabidopsis, *SRK2E* is a positive regulator of ABA-induced stomatal closure and is involved in stress adaptation. Additionally, *SRK2E* acts as a transcriptional repressor involved in the inhibition of plant growth under abiotic stress conditions [67]. In this study, the *SRK2E* gene was upregulated in RRIM600 after 90 min of cold treatment.

The *phototropin* (*PHOT1*) gene, which is a blue-light receptor kinase that optimizes photosynthetic activity by sensing temperature and controlling stomatal opening [68, 69], was downregulated at 90 min and 12 h in RRIM600 relative to GT1. We also observed that after 24 h of cold stress, RRIM600 (RRIM600 24 h X GT1 24 h) showed downregulation of a gene with high similarity to *zeaxanthin epoxidase*, which plays an important role in the xanthophyll cycle and alleviates the excitation pressure on the PSII reaction to divert photon energy into heat via zeaxanthin [68, 70].

According to our results, RRIM600 compared to GT1 exhibits dowregulated genes related to photosynthetic activity, which could affect the photosynthetic activity of this clone. Moreover, RRIM600 compared to GT1 upregulates genes related to stomatal closure. These findings are in accordance with physiological studies previously performed in rubber trees, suggesting that RRIM600 performs an “avoidance” strategy by downregulating photosynthetic activity and fast stomatal closure [10].

### Molecular markers and alternative splicing for rubber tree breeding

Molecular markers such as SSRs and SNPs are abundant in plant genomes. The development and genotyping of molecular markers is an important tool in genomic breeding and is the basis for genome selection (GS), genetic mapping and genome-wide association mapping (GWAS), as well as genetic linkage mapping. Next-generation sequencing allows the identification of thousands of putative SSR and SNP markers. Additionally, the identification of SNPs in genes using RNA-seq data allows the development of markers in candidate genes and the investigation of the variability of these genes within rubber tree clones [13].

In this study, we identified a total of 27,111 putative SSRs and 264,341 putative SNPs. The putative molecular markers identified in this study can be employed as a source to develop new markers for the species. The recent release of the *H. brasiliensis* genome associated with the genome annotation can be used to evaluate gene content and develop new markers in potential candidate genes.

Although a recent version of the genome containing 7453 scaffolds was released, the rubber tree genome is 71% repetitive [19], which makes it difficult to assemble these scaffolds into chromosomal units. The development of new SSR markers can also be carried out and is an important tool helping link these scaffolds.

Recently, studies of AS events in crop species have been conducted due to the importance of AS in the formation of multiple distinct mRNAs from a single gene [33]. In addition, AS is involved in gene regulation and may regulate many physiological processes in plants, including the response to abiotic stresses such as cold stress [1, 71].

For rubber trees, previous analyses of AS events were restricted to specific genes, such as rubber particle protein membrane [72] and sucrose transporter genes [73].

A recent study in rubber trees using third-generation sequencing data with leaf tissues under normal conditions identified a total of 636 IR events corresponding to 41% of all AS [6]. The authors suggested that the number of identified AS events represented a small subset of the total possible number of AS events because their study used untreated leaf samples. In this study, we described AS events in rubber trees using leaf tissue under abiotic stress conditions for the first time. We detected a total of 9226 IR events with a minimum of 10 reads supporting the AS sites, which corresponded to 45.5% of all events identified. Alternative donor events represented a minority of the total number of detected AS events, i.e., 13%.

The results of the present study provide an overview of AS events in rubber trees based on nonstressed and cold-stressed samples. Due to the importance of AS in plant adaptation, these data can be employed for further investigation of cold-stress adaptation in this species.

### Rubber tree breeding

Compared to other crops, rubber tree domestication and breeding, which started approximately 100 years ago, are recent [13]. Recent physiological studies involving rubber tree genotypes have demonstrated that RRIM600 is resistant to cold stress, while GT1 is cold tolerant, showing little leaf damage after 18 days of chilling exposure. It has been suggested that RRIM600 presents an “avoidance” strategy, in which it rapidly closes its stomata and downregulates photosynthetic activity. GT1 is considered an intermediate tolerant genotype; this clone continues to grow and remains active with little leaf damage under cold stress [10].

The RNA-seq approach utilized in this study allowed a deep investigation of the genetic response in these rubber tree genotypes under cold stress. The DEG analysis performed in this study provides insight into the genes that were upregulated in each genotype, i.e., RRIM600 and GT1, across different time points under cold stress and corroborates the physiological findings of Mai et al. [10]. In this study, we observed at the expression level that the RRIM600 cold-stress-response strategy focuses on strengthening the ROS scavenging system more than the GT1 cold-stress-response strategy, based on the large number of genes related to ROS scavenging that were upregulated in RRIM600 during cold treatment. Considering that several important genes previously reported to be involved in stomatal closure via the ABA signaling pathway were upregulated in RRIM600 and the enrichment of genes involved in the respiratory chain, the maintenance of a strong ROS scavenging system is necessary.

In contrast, genes related to cell growth, cell wall and photosynthesis were downregulated in RRIM600 relative to GT1 under cold stress. Although this “avoidance” strategy protects the plant from the damage caused by low temperatures, it can decrease growth and reduce the production of the clone. For rubber tree breeding, a strategy balancing costs, in which the plant remains active and has efficient ROS scavenging, is necessary. This strategy might cause a decline in latex production but could be less damaging to farmers than a complete avoidance strategy. In addition, this strategy may be advantageous to young plants because they might be able to cope with cold stress and continue growing. In such a scenario, the use of rubber tree clones that exhibit the above cold-response strategy is seemingly advantageous for farmers in the rubber industry.

Rubber tree breeding programs are interested in genotypes that can endure low temperatures and that exhibit latex production that does not cease during the cold season. The elucidation of different chilling tolerance strategies linked to information about possible genes involved in such responses, including the identification of molecular markers in these genes associated with information on AS events, provides a powerful tool for the genetic and genomic analyses of the rubber tree for breeding strategies and future studies involving GWAS and GS.

## Material and methods

### Plant materials and cold-stress treatment

Plantlets of the rubber tree clones RRIM600 and GT1 at 6 months of age were provided by Centro de Seringueira, Votuporanga, Sao Paulo, Brazil. Each clone had 3 plantlets representing 3 biological replicates.

The plants were transferred to a growth chamber set to 28 °C with a 12 h photoperiod and were watered every 2 days for 10 days. After 10 days, the plantlets were exposed to chilling stress at 10 °C for 24 h. Leaf tissues were sampled at 0 h (control), 90 min, 12 h and 24 h after cold exposure. The samples were immediately frozen on dry ice and stored at − 80 °C until use.

### RNA extraction and cDNA library construction and sequencing

Total RNA was extracted using a modified lithium chloride protocol [73]. RNA integrity and quantity were assessed using an Agilent 2100 Bioanalyzer (Agilent Technologies, Palo Alto, CA). Approximately 3 μg of total RNA was employed to construct cDNA libraries using the TruSeq RNA Sample Preparation Kit (Illumina Inc., San Diego, CA, USA). Index codes were added to each sample, and the cDNA libraries were prepared following the manufacturer’s recommendations. In total, 24 cDNA libraries (3 replicates of each genotype for each time series) were prepared. Library quality was evaluated with a 2100 Bioanalyzer (Agilent Technologies, Palo Alto, CA), and the libraries were quantified via qPCR (Illumina protocol SY-930-10-10). The 24 samples were randomly pooled (4 samples per pool) and clustered using the TruSeq PE Cluster Kit on the cBot platform (Illumina Inc., San Diego, CA, USA). Subsequently, the cDNA libraries were sequenced using an Illumina Genome Analyzer IIx with the TruSeq SBS 36-Cycle Kit (Illumina, San Diego, CA, USA) for 72 bp PE reads.

### Data filtering

The raw data generated via Illumina sequencing in the BCL format were converted to the qSeq format using Off-Line Basecaller v.1.9.4 (OLB) software. We further converted the qSeq files into FastQ files using a custom script. The raw reads were split by barcode, and the barcode regions were trimmed using Fastx-Toolkit (fastx_barcode_splitter.pl) (http://hannonlab.cshl.edu/fastx_toolkit/index.html).

Filtering for HQ reads was performed using NGS QC Toolkit 2.3 [74], considering only reads with a Phred quality score ≥ 20 and a cut-off value of 70% of the read length. All reads were deposited in the NCBI Short Read Archive (SRA) under accession number SRP155829.

### Comprehensive transcriptome assembly

Initially, the reads generated from the 24 samples were combined with bark reads [13] and mapped back onto the rubber tree genome [19] (accession number: LVXX01000000) with the HISAT2 aligner [75]. The alignment results were coordinate-sorted with SAMtools [76] and used in the Trinity genome-guided assembly. Furthermore, the scaffolds obtained from the rubber tree genome were employed in ab initio genome annotation with Maker-P [77] (Additional file 1: Material) due to the lack of a public genome annotation. This annotation provided an additional dataset of predicted transcripts.

The transcripts obtained in the genome-guided and ab initio genome annotations were combined with nonredundant *H. brasiliensis* ESTs from NCBI (as in Ago 2016) and used as a dataset for alignment and assembly against the rubber tree genome [19] using the PASA v2.0 pipeline [21] with the following parameters: --ALT_SPLICE --ALIGNER blat, gmap and MAXIMUM_INTRON_LENGTH = “50,000”. PASA modeled complete and partial gene structures based on splice-aware alignment to the reference genome, detecting unique assemblies, collapsing redundant models and identifying AS events.

The transcripts obtained using PASA were filtered according to the following criteria: (1) minimum length of 500 bp; (2) transcript prediction evidence, excluding transcripts that were exclusively predicted in the genome ab initio annotation; and (3) trimming of transcripts with high identity to nonplant sequences.

The filtered transcripts were clustered based on genome mapping location and according to gene structures. This final dataset was considered the comprehensive transcriptome for further analysis.

### Functional annotation

The Trinonate v2.0.1 pipeline (https://trinotate.github.io/) was employed to annotate the transcriptome. Briefly, Transdecoder (https://github.com/TransDecoder/TransDecoder/wiki) was used to predict open reading frames (ORFs) with a minimum of 100 amino acids. Transdecoder can predict multiple ORFs in the same transcript; however, if the predicted ORFs overlap, the program maintains the longest ORF. If multiple nonoverlapping ORFs are predicted in the same transcript, all are retained in the annotation. Translated ORFs and untranslated transcripts were searched against the SwissProt/UniProt database using BLASTX and BLASTP, respectively. In addition, these transcripts were associated with Gene Ontology (GO) [78] and KEGG [79] database information. The Transdecoder-predicted proteins were also searched for protein domain homology in the Pfam database using the HMMER 3.1 tool hmmscan (hmmer.org.). All the annotations were filtered with an e-value of 1e-5 and placed into a tab-delimited file.

### Differential gene expression

Reads from each library were aligned to the reference transcriptome with Bowtie2 v.2.2.6 [80], and the estimation of gene transcript abundance was performed with RSEM v.1.2.28 [81] using a Trinity accessory script (align_and_estimate_abundance.pl). The DEG analysis was performed with limma-voom [82], which estimates precision weights based upon the expression mean-variance trend to facilitate Bayesian-moderated, weighted t-statistics [83], with at least 10 counts per million (CPM) in at least 3 samples. Three biological replicates for each condition were provided for this analysis. We considered a gene to be differentially expressed by using a false discovery rate (FDR) cut-off ≤0.05. The pairwise comparison was performed between RRIM600 and GT1 for each time series of the cold treatment: RRIM600 0 h X GT1 0 h, RRIM600 90 min X GT1 90 min, RRIM600 12 h X GT1 12 h, and RRIM600 24 h X GT1 24 h. Additionally, DGE comparison within each genotype was performed (RRIM600 0 h X RRIM600 90 min; RRIM600 90 min X RRIM600 12 h; RRIM600 12 h X RRIM600 24 h; GT1 0 h X GT1 90 min; GT1 90 min X GT1 12 h; GT1 12 h X GT1 24 h) using the same methods described above.

### Gene ontology enrichment

The DEGs identified previously were subjected to GO enrichment analysis using GOseq with an FDR cut-off of ≤0.05 [84]. The enriched terms were submitted to REVIGO [85] with a medium similarity allowed (0.7) to summarize the enriched terms.

### Putative molecular marker identification

Putative microsatellites (SSRs) were identified using the MISA (MIcroSAtellite) script (http://pgrc.ipk-gatersleben.de/misa/). SSR regions were defined as containing at least a six-motif repetition for di-, tri-, tetra-, penta- and hexanucleotides.

The identification of putative SNPs was performed for each genotype. The reads obtained in this study were mapped against the reference transcriptome with bwa-mem [86] following default parameters. SAM files were converted into BAM files using SAMtools. Additionally, we used SAMtools to sort mapped reads and remove unmapped reads. PCR duplicates were removed with Picard (http://broadinstitute.github.io/picard). Freebayes software [87] was used to call variants in each processed BAM file with the following parameters: --min-alternate-count 5 --min-mapping-quality 30 --min-base-quality 20. VCFtools [88] was used to select biallelic SNPs, remove indels, and perform filtering with a minimum genotype quality of 20, minimum depth of 10 reads and SNP and mapping quality of 20.

### Quantitative RT-PCR (qRT-PCR) validation

To validate the DEG analysis, a total of 20 genes were randomly selected. The primer pairs used in the qRT-PCR analyses (Additional file 2: Table S1) were designed using Primer3 Plus software (http://www.bioinformatics.nl/cgi-bin/primer3plus/primer3plus.cgi) with the qPCR parameters. cDNA synthesis was performed by the Quantitect Reverse Transcription kit (Qiagen Inc., Chatsworth, CA, USA) using 500 ng of total RNA. The cDNAs were then diluted 1:5, and 2 μl from each sample was aliquoted for qPCR. The qPCR assays were carried out with iTaq Universal SYBR® Green Supermix (Bio-Rad Laboratories Inc., Hercules, CA, USA) following the manufacturer’s instructions and using 3 μM primer**.** The qPCR assays were performed using the CFX384 Real-Time PCR Detection System with the following cycling conditions: 95 °C for 10 min, followed by 40 cycles of 95 °C at 30 s and 60 °C at 1 min.

All qPCR experiments were performed using three technical and three biological replicates, with the exception of RRIM 600 at 0 h and 12 h of treatment, for which only two biological replicates were included due to the lack of RNA for one of the biological replicates. The *DEAD box RNA helicase* (*RH2b*) and mitosis protein (*YSL8*) genes were used as internal controls. To confirm the presence of a single amplicon of the PCR product, melting curve analysis was performed with temperatures ranging from 65 °C to 95 °C in increments of 0.5 °C. The Cq values and baseline were determined with CFX Manager 2.1 software (Bio-Rad Laboratories, Inc., USA). The primers used in this study are described in Additional file 2: Table S1. The results are presented as the mean ± standard error of the mean (SEM). Statistical significance differences (*p* < 0.05) were represented according to Student’s t-test.

### Alternative splicing identification

The filtered transcripts and AS events defined by PASA were processed using an in-house pipeline. This pipeline identifies and reclassifies AS events by simultaneously encompassing alternative 5′ and 3′ splice sites [33]. Furthermore, a minimum of 10 reads mapped at the splice junction was set as the threshold for considering an AS event.

## Additional files

Additional file 1: Material Rubber tree genome annotation. (DOCX 165 kb)

Additional file 2: **Table S1.** qPCR primer sequences of the 20 amplified genes. (XLSX 13 kb)

Additional file 3: **Table S2.** Gene annotation of the DEGs identified for each time point in RRIM600. (XLS 5420 kb)

Additional file 4: **Table S3.** Gene annotation of the DEGs identified for each time point in RRIM600 and GT1. (XLSX 470 kb)

Additional file 5: **Table S4.** GO enrichment of the up- and downregulated genes in RRIM600 at each time point of the series. (XLS 96 kb)

Additional file 6: **Table S5.** Statistical summary of the results of searches for putative SSRs. (XLSX 9 kb)

Additional file 7: **Table S6.** Statistical summary of the results of searches for putative SNPs. (XLSX 8 kb)

## Abbreviations

AltA: Alternative acceptor
AltD: Alternative donor
AS: Alternative splicing
BP: Biological Process
CC: Cellular Component
DEG: Differentially expressed gene
ES: Exon skipping
EST: Expressed sequence tag
GO: Gene Ontology
HQ: High quality
IR: Intron retention
KEGG: Kyoto Encyclopedia Gene and Genomes
MF: Molecular Function
PE: Paired-end
PSII: Photosystem II
qRT-PCR: Quantitative real-time PCR
RNA-seq: RNA sequencing
ROS: Reactive oxygen species
RuBisCO: Ribulose-1,5-bisphosphate carboxylase/oxygenase
SALB: South American leaf blight
SNP: Single nucleotide polymorphism
SSR: Simple sequence repeat
