# Supplementary material for: Deep expression analysis reveals distinct cold-response strategies in rubber tree (Hevea brasiliensis)
Source: BMC Genomics. 2019 Jun 4;20:455. doi: 10.1186/s12864-019-5852-5 (PMC6549365; doi:10.1186/s12864-019-5852-5)
Supplement: Supplementary file 1 — Material Rubber tree genome annotation. (DOCX 165 kb) [file 12864_2019_5852_MOESM1_ESM.docx]

Supplementary Material

Deep Expression Analysis Reveals Distinct Cold-response Strategies in the Rubber Tree (Hevea brasiliensis)

Camila Campos Mantello, Lucas Boatwright, Carla Cristina da Silva, Erivaldo Jose Scaloppi Junior, Paulo de Souza Goncalves, Brad Barbazuk, Anete Pereira de Souza^*^

*** Correspondence:** anete@unicamp.br

# Supplementary Data

**Rubber Tree Genome Annotation**

The 7,453 scaffolds from the rubber tree genome were filtered in order to selected scaffolds ≥ 3 kb. A total of 5,418 scaffolds were selected, representing 99.7% of the genome, and annotated using MAKER-P [1]. The *ab initio* annotation was performed using proteins and transcripts from the (1) *H. brasiliensis* database, (2) *Arabidopsis thaliana* (TAIR10) and *Manihot esculenta* (v6.1) to support the predictions of orthologous genes (Figure 1).

The database of *H. brasiliensis* consisted of 39,932 nonredundant and trimmed NCBI ESTs (as of June/2016), and an annotation was generated by cufflinks (ref) using *default* parameters. The cufflinks annotation was obtained using the following steps. (1) The filtered reads obtained from leaves isolated in this study and bark (SRX371361) were aligned using HiSat2 (*default* parameters) against the scaffolds ≥ 3 kb. (2) The bark and leaf filtered reads were used to generate *de novo* and genome-guided assemblies using Trinity with the default parameters. The transcripts obtained in both assemblies were filtered, and transcripts with sizes between 1 to 10 kb were selected because they potentially represented full-length transcripts (Figure 1). The trimmed ESTs were also used for PASA alignment (Figure 1). The PASA annotation file containing the gene structure was used along with the leaf and bark aligned reads and cufflinks software to update the annotation file.

The cufflinks updated annotation file, the transcripts and proteins from *M. esculenta* and *A. thaliana*, and the vector-cleaned ESTs from NCBI were combined to assist the *ab initio* gene prediction. The software SNAP [2] and Augustus [3] in MAKER-P were chosen to predict genes. The options est2genome, which uses evidence from the rubber tree transcripts, and protein2genome, which searches genes based on protein homology, were also used to improve the predictions. The option to detect alternate splicing events was also enabled.

A total of 138,605 genes containing 167,749 transcripts/proteins were predicted. The N50 was 2,340 bp. The total gene structures (exons, introns and UTRs) represented 347 Mb from the genome, corresponding to 25% of the total genome that was annotated (Table 2). The transcripts were annotated via BLASTX using the SwissProt/UniProt protein database with an evalue of 1E10-5. In total, 112,455 (67%) transcripts were annotated, of which 52,470 were annotated as transposable elements. The transcripts containing high similarity with transposons were excluded from further analysis. Additionally, transcripts ≤ 100 bp were also excluded. Of a total of 114,338 transcripts, 59,985 were annotated with the SwissProt/UniProt database and subsequently used to assist the assembly of the comprehensive transcriptome.


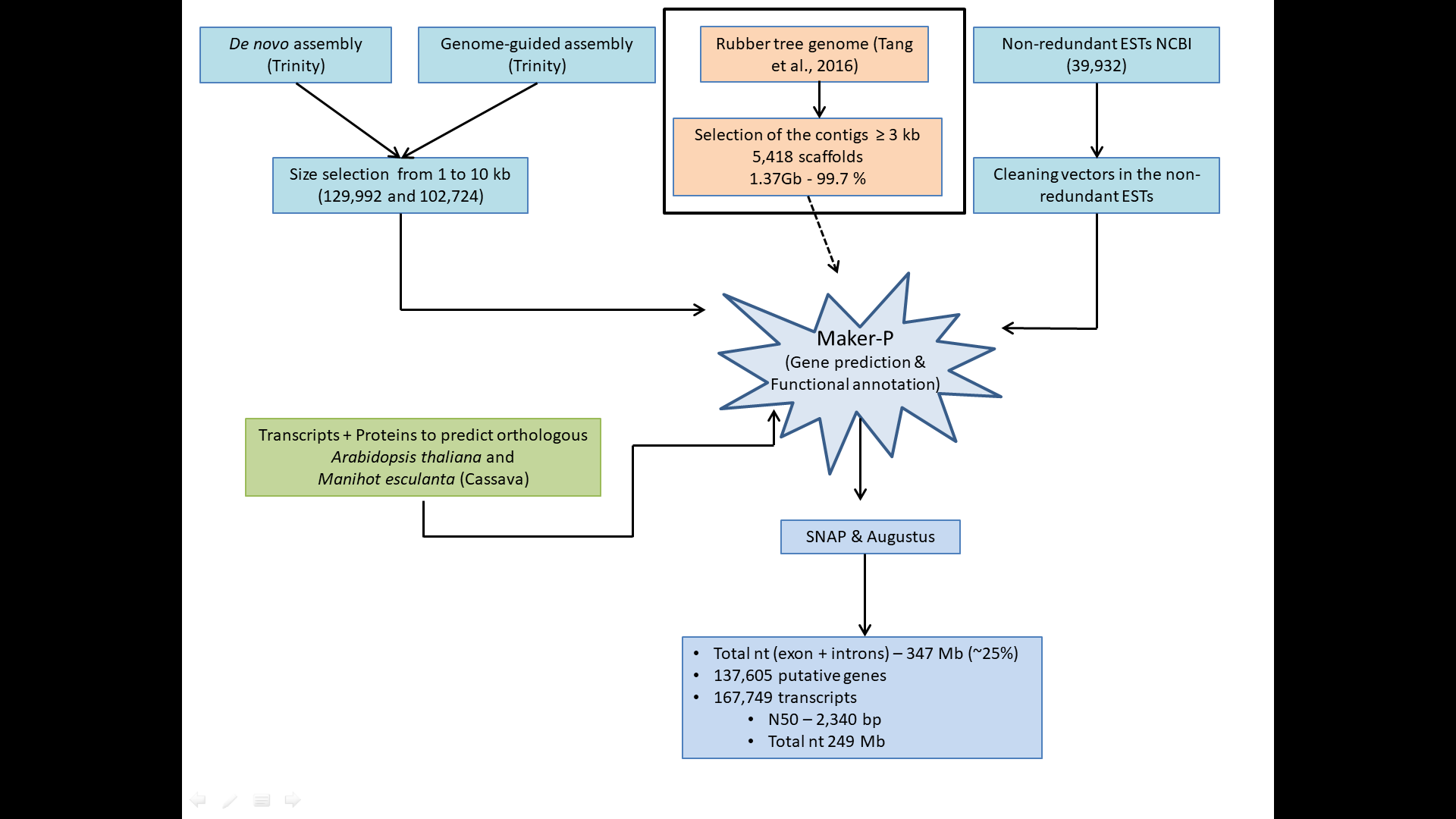


**Figure 1.** Workflow used for rubber tree genome annotation.

**References**

[1] Campbell MS, Law MY, Holt C, Stein JC, Moghe GD, Hufnagel DE, et al. MAKER-P: a tool kit for the rapid creation, management, and quality control of plant genome annotations. Plant Physiol. 2014;164:513–24. doi:10.1104/pp.113.230144.

[2] Korf I. Gene finding in novel genomes. BMC Bioinformatics. 2004;5:59. doi:10.1186/1471-2105-5-59.

[3] Stanke M, Schöffmann O, Morgenstern B, Waack S. Gene prediction in eukaryotes with a generalized hidden Markov model that uses hints from external sources. BMC Bioinformatics. 2006;7:62. doi:10.1186/1471-2105-7-62.

[4] Tang C, Yang M, Fang Y, Luo Y, Gao S, Xiao X, et al. The rubber tree genome reveals new insights into rubber production and species adaptation. Nat Plants. 2016;2:16073.
